# Genetic identification and molecular modeling characterization reveal a novel *PROM1* mutation in Stargardt4-like macular dystrophy

**DOI:** 10.18632/oncotarget.22343

**Published:** 2017-11-09

**Authors:** Saber Imani, Jingliang Cheng, Marzieh Dehghan Shasaltaneh, Chunli Wei, Lisha Yang, Shangyi Fu, Hui Zou, Md. Asaduzzaman Khan, Xianqin Zhang, Hanchun Chen, Dianzheng Zhang, Chengxia Duan, Hongbin Lv, Yumei Li, Rui Chen, Junjiang Fu

**Affiliations:** ^1^ Hunan Normal University Medical College, Changsha, Hunan, China; ^2^ Key Laboratory of Epigenetics and Oncology, Research Center for Preclinical Medicine, Southwest Medical University, Luzhou, Sichuan, China; ^3^ Chemical Injuries Research Center, Baqiyatallah Medical Sciences University, Tehran, Iran; ^4^ Laboratory of Neuro-organic Chemistry, Institute of Biochemistry and Biophysics, University of Tehran, Tehran, Iran; ^5^ Laboratory of Systems Biology and Bioinformatics, Institute of Biochemistry and Biophysics, University of Tehran, Tehran, Iran; ^6^ State Key Laboratory of Quality Research in Chinese Medicine, Macau University of Science and Technology, Macau, China; ^7^ The Honors College, University of Houston, Houston, TX, USA; ^8^ Department of Molecular and Human Genetics, Baylor College of Medicine, Houston, Texas, USA; ^9^ Department of Biochemistry, School of Life Sciences & the State Key Laboratory of Medical Genetics, Central South University, Changsha, Hunan, China; ^10^ Department of Bio-Medical Sciences, Philadelphia College of Osteopathic Medicine, Philadelphia, Pennsylvania, USA; ^11^ Department of Ophthalmology, First Affiliated Hospital of Southwest Medical University, Luzhou, Sichuan, China

**Keywords:** stargardt disease-4 (STGD4), next generation sequencing, PROM1, missense mutation, molecular modeling

## Abstract

Stargardt disease-4 (STGD4) is an autosomal dominant complex, genetically heterogeneous macular degeneration/dystrophy (MD) disorder. In this paper, we used targeted next generation sequencing and multiple molecular dynamics analyses to identify and characterize a disease-causing genetic variant in four generations of a Chinese family with STGD4-like MD. We found a novel heterozygous missense mutation, c.734T>C (p.L245P) in the *PROM1* gene. Structurally, this mutation most likely impairs PROM1 protein stability, flexibility, and amino acid interaction network after changing the amino acid residue Leucine into Proline in the basic helix-loop-helix leucine zipper domain. Molecular dynamic simulation and principal component analysis provide compelling evidence that this PROM1 mutation contributes to disease causativeness or susceptibility variants in patients with STGD4-like MD. Thus, this finding defines new approaches in genetic characterization, accurate diagnosis, and prevention of STGD4-like MD.

## INTRODUCTION

Stargardt disease (STGD) is a common, heterogeneous juvenile-onset macular degeneration/dystrophy (MD) disease with an estimated incidence of 10-12.5 per 100,000 people [1, 2]. Clinically, STGD is usually characterized by decreased central vision, night blindness, mildl atrophy of the macular photoreceptor cells, underlying retinal pigment epithelium, and gradual appearance of fundus flecks in the posterior pole of the retina [3]. This STGD disease is mostly inherited as an autosomal recessive trait of STGD (arSTGD) while Stargardt disease-4 (STGD4) is an autosomal dominant trait (adSTGD), which is associated with mutations in various genes. STGDs are mutagenetically categorized in three types/genes: *ABCA4* gene mutation (1p22.1) (STGD1 [MIM248200]) [4-6], *ELOVL4* gene mutation (6q14.1) (STGD3 [MIM600110]) [2, 7, 8], and *PROM1* gene mutation (4p15.32) (STGD4 [MIM603786]) [9-11]. Most recent families of MD and adSTGD-like genes were mapped and described in the STGD4 locus of patients with retinitis pigmentosa (RP) [10, 12].

PROM1 (Gene ID: 8842, also named as PROML1, CD133 and AC133) encodes prominin (mouse)-like 1 protein Prominin-1 containing 97 kDa protein with 865-amino acid. As a topic glycoprotein, PROM1 constitutes a 5-transmembrane domain glycoprotein with two short N (extracellular)- and C (cytoplasmic)-terminal tails and two large N-glycosylated extracellular loops in both rod and cone photoreceptor cells. Prominin-1 has been expressed originally in ependymal cells, human fetal spinal cords, and the oligodendroglia [13, 14] and is a well characterized biomarker of normal and cancerous stem cells in the central nervous system [15]. PROM1 is positioned at the apical surface of retinoblastoma cell lines and adult retina [16, 17]. Current scientific literatures have indicated that the PROM1 is likely to be involved in the protrusion, organization, and cholesterol composition of the plasma membrane [18]. In addition, PROM1 plays crucial roles in the biogenesis of photoreceptor rod discs concentrated in plasma membrane evaginations of the nascent disc membranes. Mutations in the *PROM1* gene have also been reported in other MD diseases, such as STGD4 [10, 12, 19] and cone-rod dystrophy [9-11], which demonstrates that mutations in same gene may manifest multiple clinical phenotypes.

In STGD4-like MD, defective PROM1 is similarly involved in plasma membrane invaginations and disk malfunction, particularly in the myoid region of the photoreceptor [10, 20]. In the photoreceptor cells of STGD4-like MD, PROM1 cannot be migrated into the outer segment where disks are formed, leading to disk malfunction and vision problem [21]. Even with developing knowledge, the function and mediator roles of PROM1 in MD disease are not well characterized. Despite numerous genetic studies focused on identifying of *PROM1* mutation (mainly frameshift) in Pakistani [20], Saudi Arabian [22], Spanish [9], and Indian [18] families with MD degeneration, the molecular roles of these mutations are still obscure. An interesting area to continue research is the specific mechanism of mutations on the *PROM1* gene in the disk morphogenesis, as well as how this misallocation of disks in photoreceptors could be transmitted as an autosomal dominant trait and/or as an autosomal recessive trait.

In this study, we considered a Chinese family with STGD4-like MD that has a novel, deleterious, pathogenic and disease-causing mutation, c.734T > C (p.L245P), in the *PROM1* gene. The structural, molecular dynamics and energetic analysis of the wild and mutational types of PROM1 gave us a clear overview of the fundamental process at the molecular level. A combination of molecular dynamic simulations and principal component analysis (PCA) is applied to investigate the molecular mechanism of the mutation’s conformational diversity. Free energy landscapes (FEL) based on trajectories of the simulation also provide detailed information related to be conformational changes of proteins. Thus, FEL using multiple physical parameters from the molecular simulation trajectory are calculated to reveal related energetic information of the mutation’s conformational diversity.

## RESULTS

### Pedigree information and clinical findings

**Figure 1:**
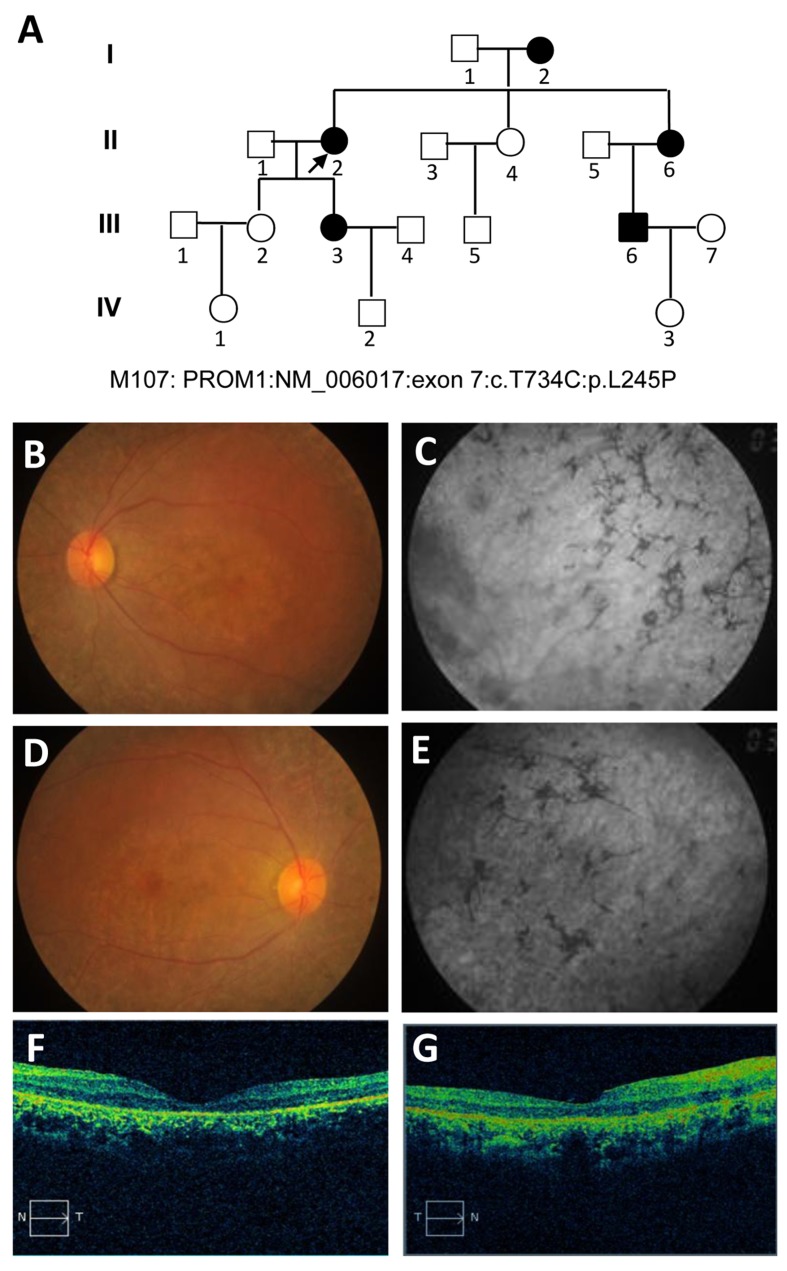
Pedigree of the M107 family with autosomal dominant Stargardt4-like macular dystrophy and clinical assessment of affected proband **A.** Pedigree of the M107 family. Family number and disease-causing mutation (s) are noted at this pedigree. Normal individuals are shown as clear circles (females) and squares (males), affected individuals are shown as filled symbols. The patient above the arrow indicates proband (II: 2) (molecular no.: M107). Individuals with heterozygous mutation are represented in filled symbols. Targeted next generation sequencing individual indicated with arrow. **B.**, **D.** Fundus photography of the left and right eyes, respectively. **C.**, **E.** Fundus autofluorescences of left and right eyes, respectively. **F.**, **G.** OCT characterization of the left and right eyes with conclusive genetic defects, respectively. The macular progressive depigmentation with atrophy and pigment clumping are major complement of proband.

A four-generation pedigree (M107, proband: II: 2) of a Chinese family, in which one or more members have a mixed retinal dystrophy, was created in the study (Figure 1A). The proband, 63 years old women, claimed to have issues with sight ever since the age of 42 with bilateral Bull’s eye maculopathy. The images of the proband are documented by fundus photography (FP), fundus autofluorescence (FA), electroretinograms (ERGs), and optical coherence tomography (OCT), which are shown in Figure 1B-1G, respectively. As the FA results illustrated, the absence of a normal darkened presence, replacement of a central reddish orange hue and close mottling (especially in the left eye, Figure 1B-1C), bilateral macular discoloration, and peripheral mottling with intraretinal pigment clumping (especially in the right eye, Figure 1D-1E) are major abnormalities in the retinal examination. The FA showed dark choroid with limited staining of some white fundus flecks in both eyes. Our results revealed possible chorioretinal atrophy in the right eye, which is highlighted by perifoveal and parafoveal hypofluorescent (Figure 1E). The outer retinal architecture in left and right eyes was lost, respectively (Figure 1F-1G), which was extremely apparent in the macula. Importantly, macular progressive depigmentation with pigment clumping and atrophy is a major complement of the proband.

Other patients showed similar clinical and ophthalmological abnormalities. Tunnel vision and loss of peripheral vision were apparent in all patients. The presenting symptoms for proband and pedigree II:6 were more severe, especially in night blindness. Based on our pedigree analysis in the proband, there were decreased central vision and visual acuity, and a prominent presence of fundus flecks in the posterior pole of the retina, which demonstrated a late onset STGD4-like MD. Neither STGD4-like MD nor MD-related ophthalmological defects was observed in other recruited normal family members.

### Mutation identification and segregation analysis

**Figure 2:**
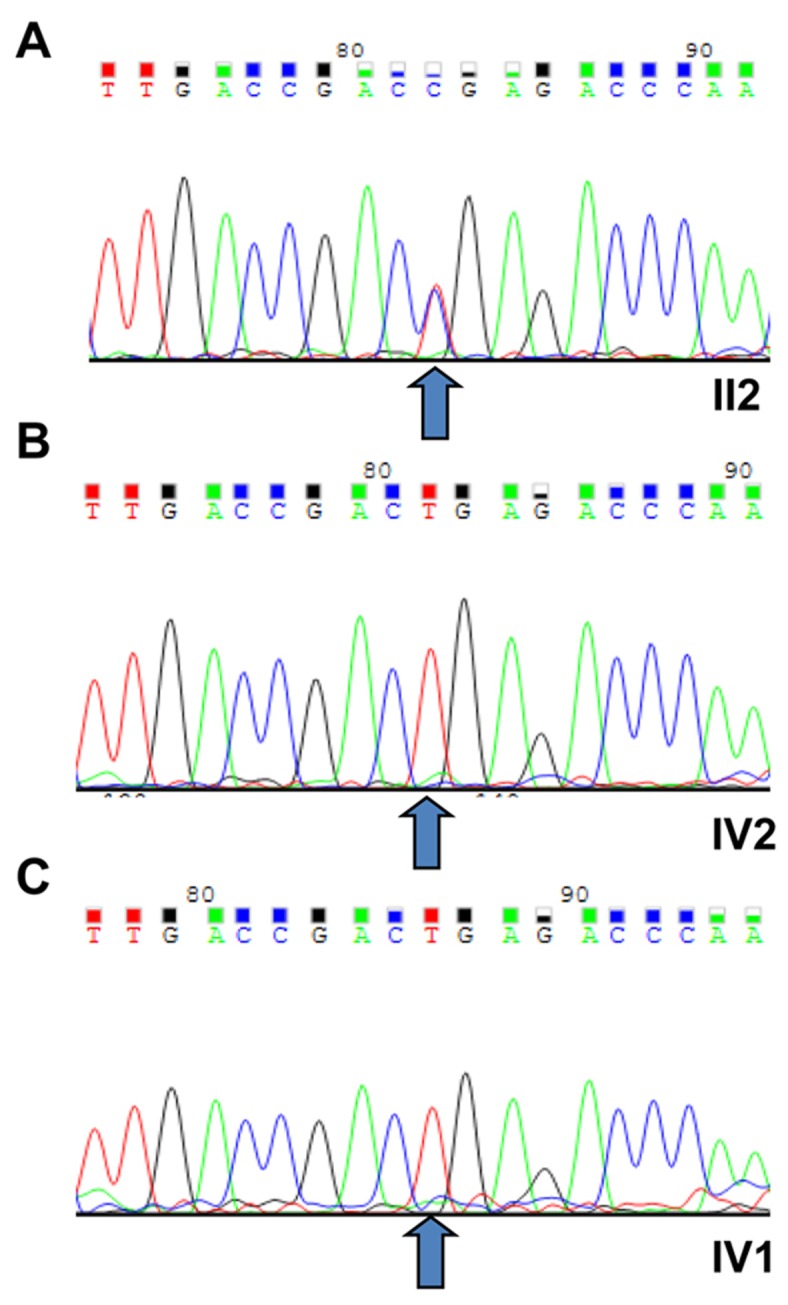
Sequencing results of the (c.T734C:p.L245P) *PROM1* mutation **A.** The novel heterozygous missense mutation (PROM1: NM_006017: exon7: c.T734C:p.L245P) of *PROM1* gene in proband (II:2). **B.** Sanger sequencing results of IV:2 identified as a wild type. **C.** The wild type IV:1 was validated in the unaffected family individuals and unrelated normal controls, with the normal male from no eye disease history family (III:1). All number were depicted in Figure 1. The arrows indicate the mutation at the nucleotide position c. 734T > C in the *PROM1* gene.

Targeted exome sequencing (TES) was analyzed using a genomic DNA (gDNA) sample of the proband (pedigree II:2) from the STGD4-like MD affected Chinese family. In total, more than 10 million bases of the sequence with a 100-bp read length were generated, and 99% of the bases passed the quality assessment. However, 40,000 single nucleotide polymorphisms (SNPs) and Insertions/Deletions (INDELs) were found. Then, 99% of the billion bases were aligned with the human reference sequence, which had billions of bases covered by a 40-fold coverage target region. As the data was filtered, we excluded known variants recorded in dbSNP137, 1000 genomes project, and HapMap with minor allele frequency (MAF) > 0.50%. Next, the missense mutations in the *PROM1* gene were selected for segregation analysis and conformational Sanger sequencing (Figure 2). We identified a novel heterozygous missense mutation (c.734T > C) of *PROM1* gene (NM_006017) (depicted in Figure 1A with pedigree II:2 and IV:2, respectively; Figure 2A-2B), leading to a change of amino acid from Leu to *Pro* at the 245^th^ position of PROM1 protein (p.L245P). The c.734T > C variant was consequently validated based on its absence in unaffected family individuals and unrelated normal controls (100 individuals), which consisted of normal people without a family history of eye disease (wild type, depicted in Figure 1A with pedigree IV:1; Figure 2C). This variant was confirmed by Sanger sequencing and it showed complete segregation in this pedigree M107 family association furthering population studies and pinpointing its role in STGD4-like MD pathogenesis.

### The predictions of conservation and damage in the c.734T > C (p.L245P) mutation on the PROM1 protein

The PROM1 protein alignment analysis based on multiple sequence alignment in different organisms’ genes is shown in the Supplementary Figure S1. The nucleotide change results in a Pro substitution for a highly conserved Leu at residue 245 (p.L245P). The PROM1 p.L245P mutation in a basic helix-loop-helix leucine zipper domain (bHLH-Zip) indicates an important role in transcriptional regulation of PROM1 in patients with STGD4-like MD, thereby contributing to disease progress. Computer-based protein analysis in different species indicates that this variant is likely a pathogenic mutation (Table 1). The damaging consequence on protein indicates that the variant in *PROM1* gene was probably “disease-causing” with a score of 1 on Polyphen-2 and 0.01 score in scale-invariant feature transform (SIFT) prediction analysis (sensitivity: 0.01; specificity: 1.00). Also, Mutation Taster predicted that the PROM1 heterozygous mutations c. 734T > C (P.L245P) is functionally ‘‘damage-causing’’ (Mutation Taster score is 98). I-Mutant3.0 (stability predictors, DDG < 0 (decrease stability) and DDG > 0 (increase stability)) showed the variant decreased stability with a score of -1.59 kcal/mol (Table 1).

**Table 1:** Characteristics of PROM1 SNP in Chinese family with adSTGD and analysis of their predicted protein structure and disease-causing effects.

Exon	Variation				Polyphen-2	Mutation Taster	I-Mutant3.0	SIFT	EXAC
	Nucleotide*	Protein*	Type	Status					
7	c.734 T>C	P.L245P	Missense	Heter	PD (1.000)	DC (98)	DS (-1.59 kcal/mol)	D (0.01)	Novel

Abbreviations: c, variation at cDNA level; p, variation at protein level; L245P, Proline substitution conserved leucine at codon 245; Heter, heterozygote; PD, probably damaging; DC, disease causing; DS, Decrease stability; D, damaging.

* All nucleotide and amino acid abbreviated according the International Union of Pure and Applied Chemistry (IUPAC).

### Quality check of constructed model

Our homology modeling results were evaluated to choose the best constructed model for other processes. The overall quality of the model was further checked using ProSA-web server, then the Z-score of the models were calculated. The 3D model was accepted based on the standards of the ProSA-web server. The ProSA-web Z-score is an indicator of the overall quality of a model. In this regard, ProSA-web Z-score of the studied model was negative and within the range of -4 to -4.30, indicating it has desirable qualities (Supplementary Table 1). Further confirmation of the 3D models was performed using quantitative model energy analysis (QMEAN) tools, supporting the quality for the model (Supplementary Table 1). Scoring function was assessed based on geometrical analysis using QMEAN Z-score for all modeled structures. This result shows the quality of constructed model based on the template including 0.3 to 0.4.

### Results of molecular dynamic simulation analysis

**Figure 3:**
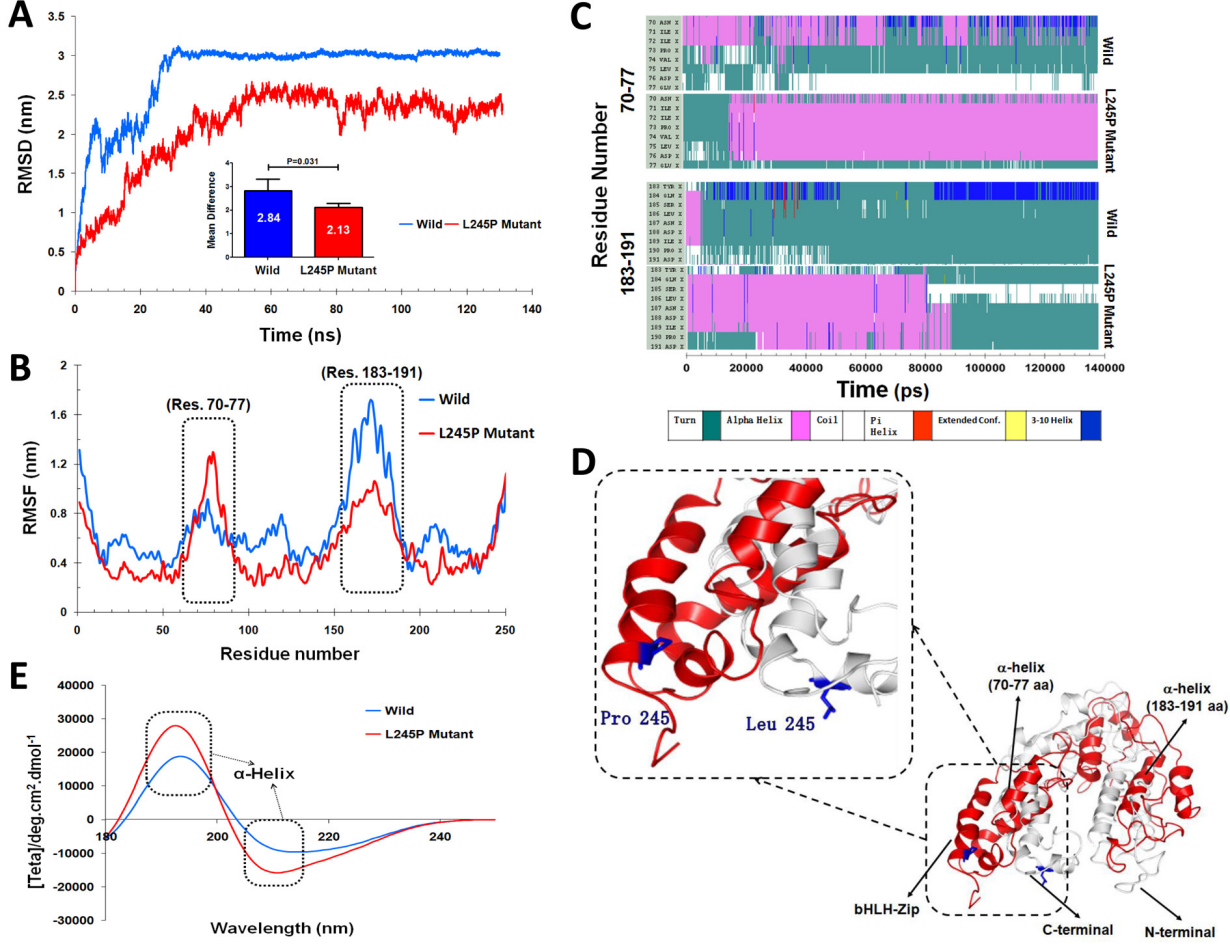
Functional effects of the c.734T > C mutation on the PROM1 protein **A.** RMSD plots of the wild-type and the L245P complex systems for 130 ns of simulation, where time step is plotted on X-axis while RMSD (nm) is plotted on Y-axis. **B.** Difference in average Cα-RMSF of the L245P mutant (red) and the wild type (blue). The maxima differences of residue-level displacements and fluctuations of both systems are shown in the residues numbers 70-77 and 183-191. **C.** The secondary structure as a function of the simulation time for two model structures. Upper and lower panels indicate secondary structure for the wild-, and -mutant type in the two main functional extracellular domains of PROM1 protein, 70-77 (upper panel) and 183-191 (lower panel) amino-acid residues, respectively. This finding indicates the initial structure of PROM1 in the wild type showing as a turn structure while the L245P mutant converted to an alpha helix during the simulation time (residues number 70-77) (upper panel). Secondary structure analysis in the residues number 183-191 shows this position including a turn conformation at the end of simulation in wild type, whereas the L245P mutant depictes an alpha-helix during 90 ns and all structures finally returned to the initial conformation at the end of simulation times. **D.** 3D structure from the wild type and the L245P mutant of PROM1 represent in gray and red color, respectively. Substitution of the Leu with Pro changes the α-helical structure of bHLH-Zip domain. The position of the p.L245P mutation is reported in blue color on the both structures. **E.** Far-UV CD spectra of secondary structure analysis of the wild and the mutant PROM1 during 130 ns-MD simulations. The two minima at 195 and 210 nm indicate an α-helical structure.

To evaluate the structural deviation of both wild and mutated residue during the simulation, distributions of root mean square deviation (RMSD) of backbone atoms were calculated. We investigated the structural and functional consequences of the reported deleterious point mutation as an active site domain of PROM1 during a 130 nanosecond (ns) molecular dynamic simulations phase (Figure 3). The RMSD values of the PROM1 backbone atoms in the entire molecular dynamic simulation trajectory (130 ns) are shown in Figure 3A. As seen in this figure, the wild type equilibrates after 25 ns with the structure maintaining a level of 30 Å, whereas the mutant variant shows a significant decrease (*P* = 0.031) in the RMSD value with more fluctuations containing low stabilization, which has an average of ≈ 12 Å. During the simulation, the L245P mutant backbone atoms were aligned with RMSDs of less than 20 Å, however fluctuations were remarkably visible over the course of simulation.

We also calculated the residue-based root mean square fluctuation (RMSF) differences between each amino acid of PROM1 to directly gain insights into the structural fluctuation and flexibility of this point mutation; details are shown in Figure 3B. We found that the L245P point mutant protein has different fluctuation patterns than the wild-type protein. The RMSF graph clearly shows that mutant variant has significantly higher fluctuations in the side chain residues from number 70 to 77. To understand the nature of these structural alterations, secondary structure assignments for all protein entries were performed using a defined secondary structure of protein (DSSP) algorithm [23]. Results from DSSP analysis confirmed these regions (residues number 70-77) in the structure generated during the simulation; furthermore, it revealed that the loop in the initial human PROM1 structure folded into a small helix after the molecular dynamic simulation (Figure 3C-3D). In contrast, the simulated wild complex C-alpha atoms have more fluctuations in the 183-1910 residues number compared to mutant type (Figure 3B). This result indicates that the initial structure of PROM1 in wild type exhibits turn structure while L245P mutant is converted to alpha helix during 90 ns, before all structures returned to the initial conformation at the end of simulation times. Therefore, both RMSF and RMSD results demonstrated the L245P mutation markedly influences the structure of the active site of PROM1. Circular dichroism (CD) server was used for analyzing secondary structure of the wild-type and mutant PROM1 in the wavelength of far-UV i.e., 195-260 nm (Figure 3E). This graph clearly shows that the secondary structure of L245P mutant system is a more dominant alpha helix than that in the wild type with two minima around 195 and 210 nm, contributing to the stable structure of bHLH-ZIP domain of L245P mutant system.

**Figure 4:**
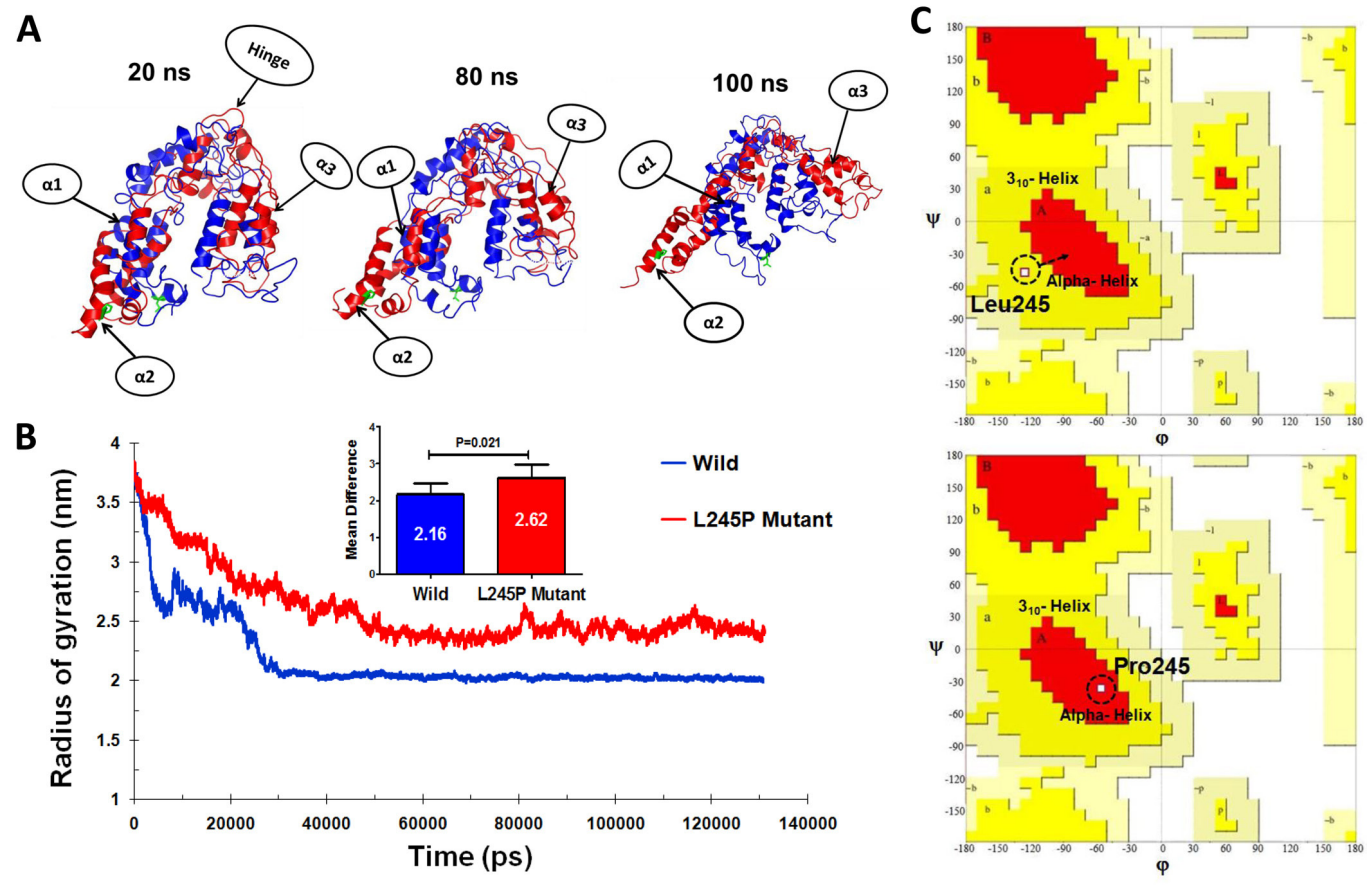
Tertiary structural stability of the (c.734T > C) p.L245P mutant PROM1 **A.** Structural superposition of the wild- (red) and the mutant-type (blue) domains extracted from the trajectories of 20, 80, and 100 ns. The mutation of L245P and the residue Leu245 of wild type are labeled in green color. The helices and hinge indicate with arrows. α1: residues number 70-77, α2: bHLH-Zip domain, and α3: residues number: 183-191. **B.** Plot of average RG values for each of the systems. RG for L245P mutant and wild type are colored as red and blue, respectively. The chart also shows differential RG values between residues of WT and mutant forms. There is a strong prospect that the L245P mutation influences the structure of the active site of PROM1, possibly by creating a further fold and destabilizing the mentioned domain. **C.** Comparative diagram depicting Ramchandran plot analysis of PROM1 protein variants in the wild type (upper panel) and the L245P mutant (lower panel) during 130 ns molecular dynamic simulations. Ramachandran plots show the phi (φ)-psi (ψ) torsion angles for the related residue number 245 of PROM1 in this structure. Leu and Pro residues are shown as square (□) and are not restricted to the regions of plots. In the upper panel, before mutation, Leu245 located in the 3_10_ helix region, whereas mutated residue (L245P) transferred to the alpha helix parts of plot in the lower panel during the simulation time.

To further study the evolution of the tertiary structural stability in a molecular dynamic biological system, we measured the structural alterations of the proteins during the simulation. For more details, the tertiary structural superposition of the wild type (red) and mutant (blue) were extracted in 20, 80, and 100 ns of simulations. In the wild-type simulation, the helices α1 (residues number 70-77), α2 (bHLH-Zip domain) and α3 (residues number 183-191) maintained their structures along the entire trajectory, whereas L245P mutant structure (compared to wild type conformation) exhibited a higher degree of flexibility, especially at position α2 and α3. It seems that this fluctuation is associated to the hinge region. In the mutant domain, it appears that helices α2 and α3 are partially folded so that they are displaced from its original position, thereby affecting the relaxation in the bHLH-Zip of the active domain core (Figure 4A). To examine the size of protein structure, we plotted the radius of gyration plot for the C-alpha atoms of protein *versus* time, shown in Figure 4B, where it is defined as the mass-weight root-mean-square distance of collection of atoms from their common center of mass. This provides an insight into the overall dimension of the protein. The Rg of each compound remains stable after 30 ns molecular dynamic simulation. The average values of the wild type and L245 mutant group are 2.16 ± 0.31 Å and 2.62 ± 0.32 Å, respectively. Considerable increases in the Rg values showed about 23% for the mutant, indicateing the stability of the mutant compared to the wild type, especially in the hinge region of bHLH-Zip. Thus, stable Rg values along the trajectories indicate that the overall packing of bHLH-Zip is maintained during simulations. These events lead to unstable moments of inertia in the mutant (Figure 4B). Another approach to analyze the protein dynamics is to examine the hydrogen bond formation between all residues, which is considered as a major force in controlling the stable conformation of protein structure. This result is shown as in Supplementary Figure 2. The mutant exhibited slightly lower total hydrogen bond interactions during 130 ns compared to the wild type. These results confirmed the instability of the overall mutant structure.

The Ramachandran plots of the wild and L245P mutant types are provided in Figure 4C and Table 2. They showed that 19.9% and 18.7% of residues of the wild and L245P mutant types were placed in the allowed regions, respectively. This difference between wild and mutant systems is not as drastic, indicating the slight stabilization of the optimized human PROM1 structure. The residues located in the favored zone were also increased from 72.9 to 74.9 during the molecular dynamic simulation. As shown in Figure 4C, the residue of the wild type (L245) falls in 3_10_ helix regions of the Ramchandran plot (upper panel), whereas, after Pro245 substitution, the new residue is transferred to the alpha helix parts of the plot (lower panel). This provides evidence to further confirm the results of DSSP and secondary structure prediction.

**Table 2:** Assessment of the Ramachandran plot of the wild and the L245P-mutant type.

	Favored region (%)	Favored a.a. number	Allowed region (%)	Allowed a.a. number	Outlier region (%)	Outlier a.a. number
Wild-Type	72.9	183	19.9	50	7.2	18
L245P Mutant	74.9	188	18.7	47	6.4	16

Abbreviations: a.a: amino acids; L245P, Proline substitution conserved leucine at codon 245.

### Structural effects on the p.L245P mutation in residue interaction networks

**Figure 5:**
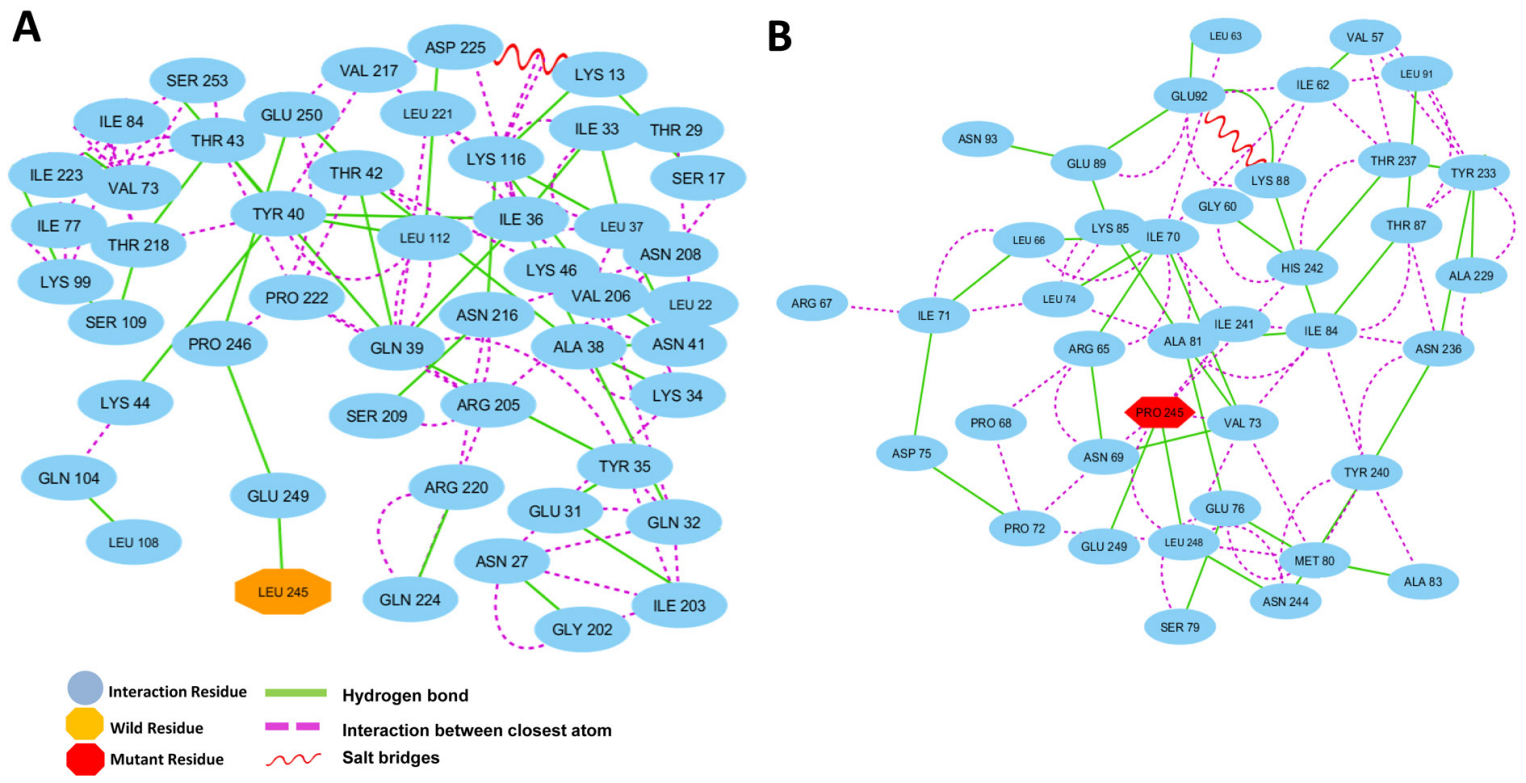
Residue interaction networks **A.** RINs of the active functional extracellular domain residues of PROM1 position in the wild type and **B.** the L245P mutant forms. Wild type reported on H-bond with Glu249 and no closely atomic interaction with the surrounding residues. L245P mutant form shows two H-bond interaction with Leu248, Glu249, and several closer atomic interactions generated through Pro245 with Ile241, His242, Asn69, Val73, and Leu248. The more interactions between the L245P mutant and neighboring residues could be destabilized at the bHLH-Zip domain in 130 ns.

The residue interaction networks (RINs) analysis of a protein backbone is a new strategy that identifies key residue interactions. The RINs assays can be used to explore the differences between different proteins, including the proteins of the wild type and mutants. In this work, we investigated the relationship between key residues of the wild type and that of the L245P mutant by generating RINs and using representative structures from 130 ns of molecular dynamics (Figure 5). Evident from the RINs plot, comparison between the predicted key residues of the wild type Leu and that of mutated Pro in the 245^th^ position revealed that the mutant residue changed the interaction within the network. In the wild type, the residue Leu245 forms one hydrogen bond with residue Glu249 (Figure 5A), whereas the mutant form exhibits that the Pro245 has the two hydrogen bonds with Leu 248 and Glu249. Also found in the mutant form of PROM1, the α-helical structure in the bHLH-Zip domain was noticeably stabilized through close atom interactions of Pro245 with Ile241, His242, Asn69, Val73, and Leu248 (Figure 5B). In contrast, there are no interactions of Leu245 with Ile241, His242, Asn69, Val73, and Leu248 in the wild-type system. As a result, analysis of RINs shows that the mutant residue changed the interaction within the network in comparison to the wild type, which made a more stabilized bHLH-Zip mutant structure in the extracellular domain of PROM1. Even so, L245P mutation affects the interactive network and the overall structure of the protein, leading to instability of the total structure of PROM1 protein. Hydrogen bonds in the overall structure of the wild type are slightly increased compared to the mutant type. Nevertheless, the specific hydrogen bond interactions in the mutated region were enhanced relative to the wild residue (Supplementary Figure 2), demonstrating that bHLH-Zip domain of the mutant type is more stable compared to the wild type.

### The p.L245P mutation of PROM1 dynamical properties increases fluctuation, flexibility and atomic density distribution

**Figure 6:**
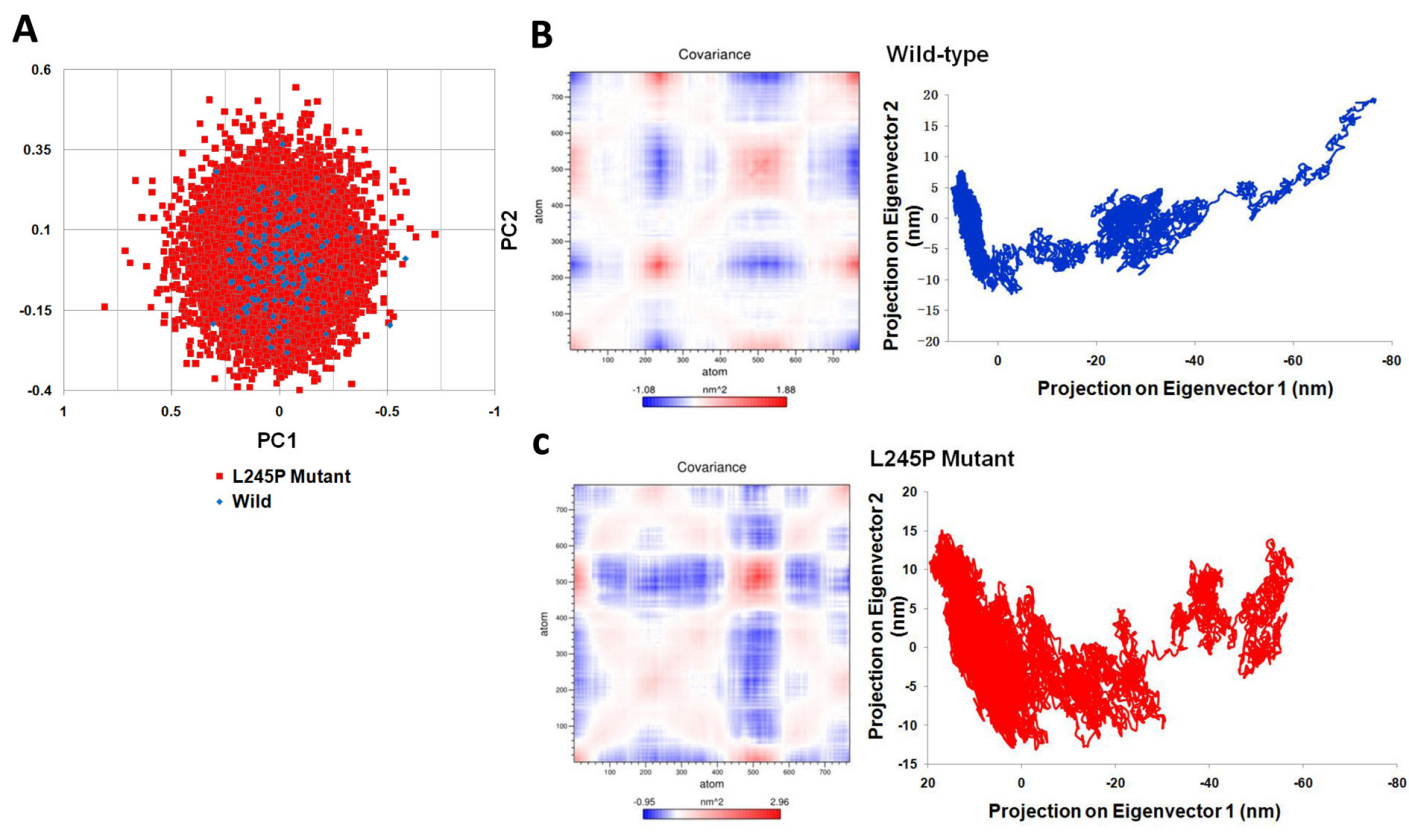
Dynamical effects of the p. L245P mutation on the PROM1 protein **A.**. PCA scatter plots along the pair of first two principal components, PC1 and PC2 for the wild-type and the L245P mutant showing differences between both types of eigenvectors. This represents optimal two-dimensional projections of the data over the 130 ns molecular dynamic trajectories. **B.** Cross correlation matrix C-alpha atomic graph and plot in during 130 ns simulation for the wild and **C.** the mutant systems. The range of motion is indicated by various colors in the panel. Red indicates positive correlation, whereas blue indicates anti-correlation. Totally, the L245P mutant effected a partial folding of the mutant protein region have the mostly anti-correlated motion; which is primarily involved in the interaction with other domains. These molecular changes were clearly depicted in the atomic density of distribution plot. There was a significant change in density distribution in native compared to the mutant. Moreover the mutant structure (L245P) shows highest atomic density distribution relative to the wild structure.

To detect main dynamical topographies of the wild-type and the mutant domains as well as highlight major conformational changes between the structures, we mathematically investigated the atomic positional fluctuations covariance matrix of the C-alpha atoms using the PCA method (Figure 6). In this study, two principal motions were considered to evaluate the general fluctuations; the first principal motion (PC1) corresponding to the scissoring motion between the interacting residues, and another one (PC2) indicateing a twisting motion. Figure 6A shows a scatter plot constructed for the wild-type and L245P mutant in both types’ eigenvectors, PC1 and PC2. As shown in Figure 6, the most fluctuations between all atoms of wild and mutant types are between -1.088 to 1.88 and -0.95 to 2.96 nm, respectively during the simulation. It means that the L245P mutant generates more scissoring motion along the PC1 axis relative to the wild type. A significant difference between these two systems are evident from the characteristic structures plotted along two principal components directions. The motions of the loop in both systems were described. PCA revealed that the L245P mutant inhibits a relatively wider phase space and shows higher fluctuation in contrast to the wild type. This result is congruent with the presented Rg graph. The molecular changes were depicted in the atomic density distribution plot (Figure 6B-6C). There was a clearly, significant change in the density distribution of the native compared to the mutant. Moreover, the mutant structure shows higher atomic density distribution relative to the wild structure. Also, the L245P mutant complex occupies a larger phase space and exhibits a higher fluctuation compared to the wild-type complex. The cross-correlation matrix and a graph of the correlated and anti-correlated motions in the wild type and mutant system are shown in Figure 6B-6C, respectively. These results indicate that a specific residue is established with all the other residues during 130 ns simulation. Specifically, the single mutation of L245P mutant causes a change in the profile of the cumulative correlation motions localized along the entire bHLH-Zip domain. This effect indicates that a partial folding of the mutant protein region has mostly anti-correlated motion, which is primarily involved in interactions with other domains.

**Figure 7:**
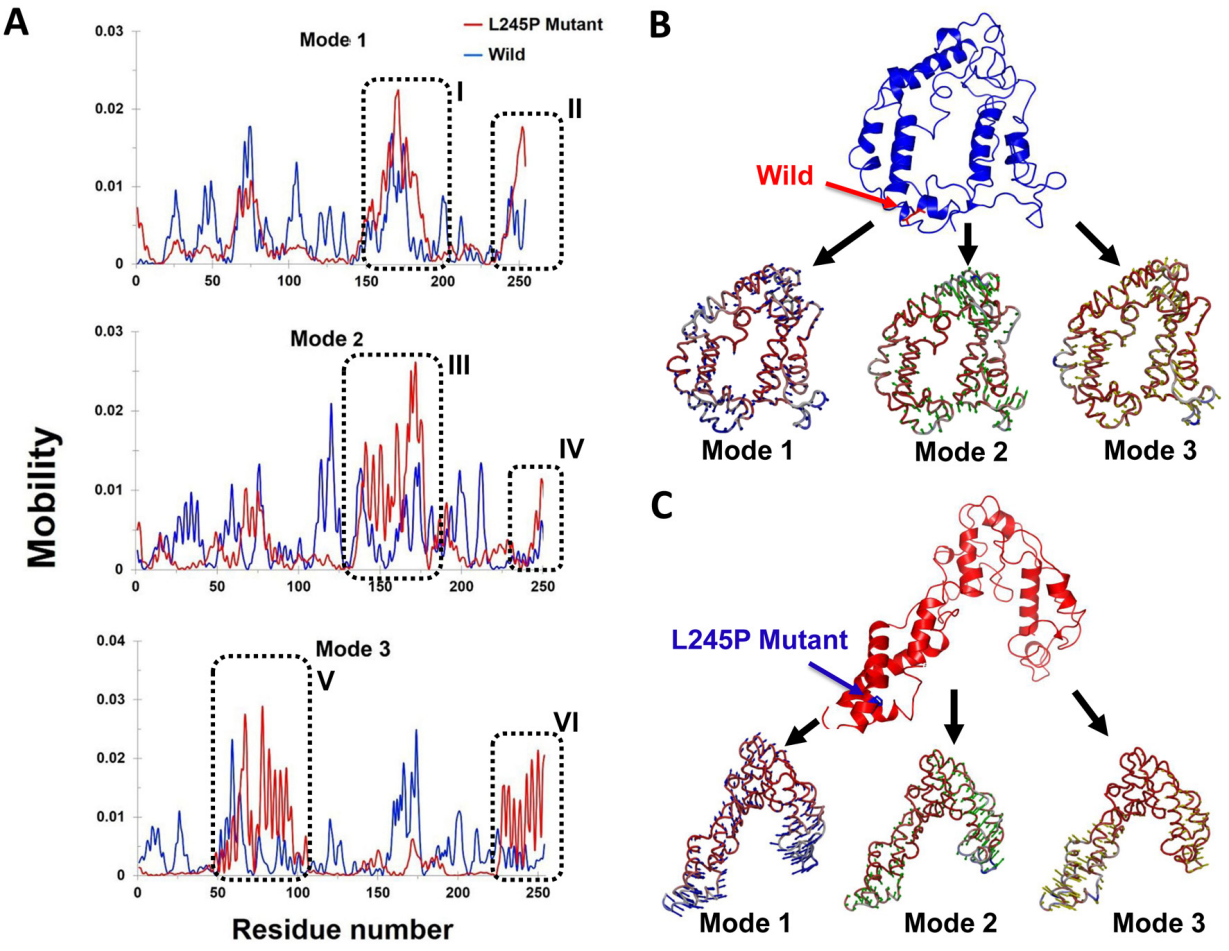
Mobility effects of the p. L245P mutation on the PROM1 protein **A.** Residue based mobility plots of the wild and mutant showing mobility at different residues across different modes. **B.** Porcupine plots in the three different modes of the wild type (blue) PROM1 protein. This graph shows the number of residues 70-77 and 183-191 playing significant roles in the secondary structure modifications of the mutant form. **C.** Motion in the three different modes of the L245P mutant PROM1 protein. Arrows in blue, green, and red indicate motions along mode 1, 2, and 3 respectively. These figures clearly show that the L245P mutation affected the overall conformational fluctuation of the system. Our results indicate the most motions in mode 1 located in residues number 183-191 **I.** and 250-255 (II) (mode 1), 120-180 (III) and 250-255 (IV) (mode 2), as well as 50-100 (V) and 230-255 (VI) (mode 3).

Illustrated by the porcupine plots, alterations in the direction of residue domain for motion could provide information about the residue dynamics of the related regions in the protein during the simulation. Generally, the first three normal modes indicate the overall motion represented by the macromolecules during a simulation. We analyzed the motion modifications between two systems i.e., wild and L245P mutant along different modes 1, 2 and 3 (Figure 7). The results show that the overall motion between two systems, as shown by the graph, is wide-ranging and different. In comparison, most motion between the wild type and L245P mutant systems was caused by residues number 183-191 and 250-255 across mode 1, graphed in the Figure 7A. A higher mobility frequency was observed in the residues number located at 120-180 and 250-255 in mode 2. Residue behavior at these positions is emphasized at the partial opening of the mutant and complete closing of all parts of the wild type. This observation was also seen in the residues number 50-100 and 230-255 of mode 3. Evidently, residues number 70-77 and 183-191 also played significant roles in the secondary structure modifications of the mutant systems. These results are consistent with the RMSF plot, in which the mentioned regions are highly vibrant compared to the other locations. Most of the motions for the wild type were due to residues number 150-180 across all three modes as shown in Figure 7B. The wild type shows a twisting motion from C-terminal to N-terminal based on the direction of the residue movement in all parts of protein. Furthermore, the direction of the above residues number points toward the center of the active site spatially in the bHLH-Zip domain, thus creating a suitable location in the membrane to launch several signaling cascades. Table 3 highlights the list of residues that attributed to the majority of motions across different normal modes in wild and mutant types. As enumerated in Table 3, the residues with the highest motion include number 70-90 and 220-250 especially at position 245^th^. These situations are more mobile than the wild type across three normal modes. These results are consistent to the Figure 4A, which showed higher motion in the α2 region i.e., bHLH-Zip domain.

**Table 3:** Prominent residue contributing towards motions of the wild and the L245P-mutant across different modes.

	Mode No.	Residue numbers with highest mobility
Wild-Type	1	71, 74, 167, 168
	2	120
	3	59, 166, 174
L245P Mutant	1	252, 170, 168, 165
	2	67, 78, 82, 83, 65, 86, 93, 228, 243, 245, 250
	3	65, 67, 78, 82, 86, 90, 228, 243, 245, 250

Abbreviations: L245P, Proline substitution conserved leucine at codon 245.

Dynamically, all these projections show that the mutant complexes have high values, signifying a large increase in the flexibility in comparison to the wild complex during the collective motion of the PROM1 protein; this pathogenic and deleterious mutation made the PROM1 protein less stable compared to the wild type protein complex (Figure 7B-7C).

### The L245P mutant induces instability in free energy landscape

**Figure 8:**
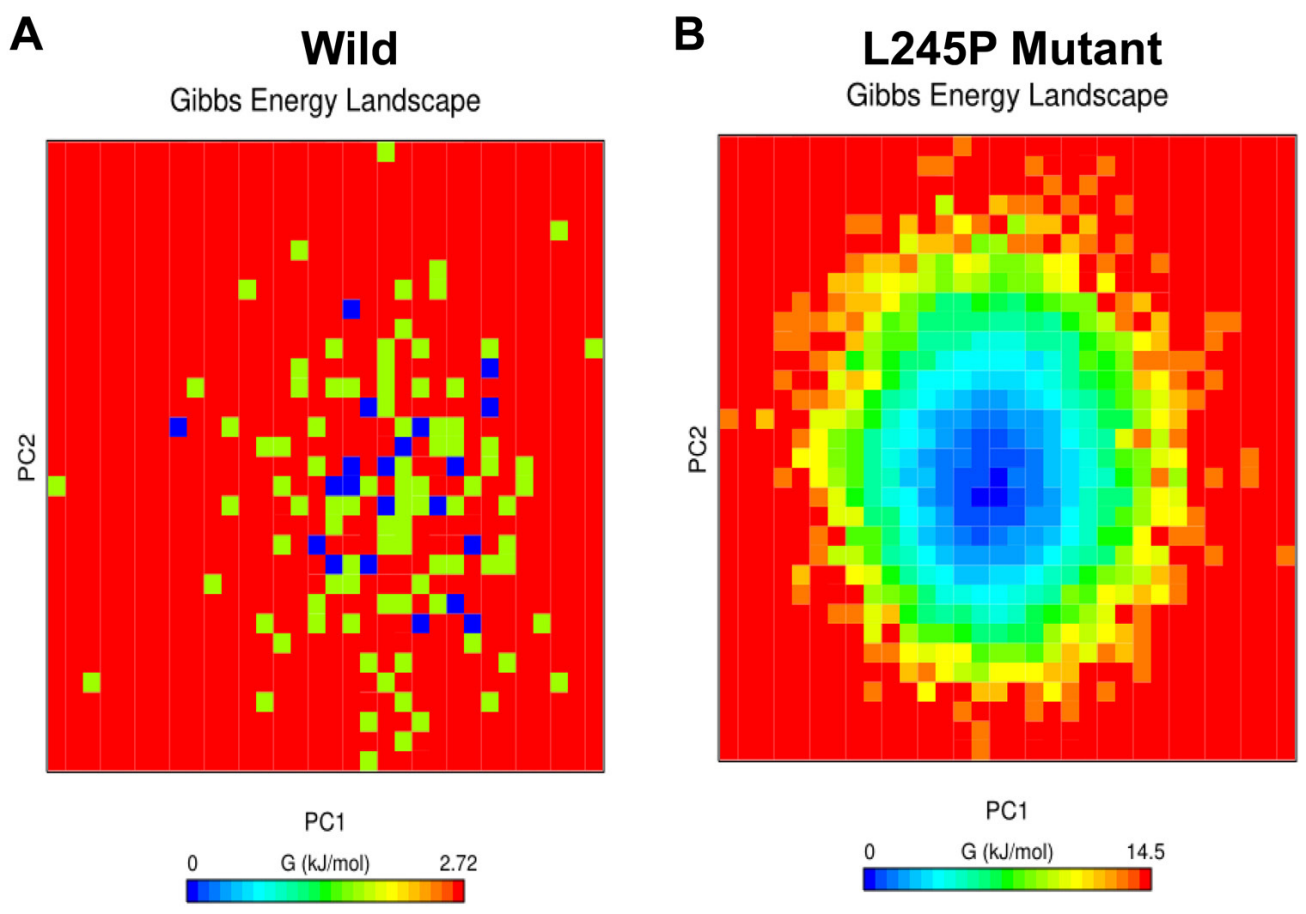
Free energy landscape (FEL) Projections of FEL of the wild **A.** and the L245P mutant PROM1 **B.** conformational space onto PC1 and PC2 produced from PCA. The dark blue indicates the lowest energy configuration and red shows the highest energy configuration. These energy levels induce the instability in the general structure of mutated protein. This observation is consistent with the PCA scatter and the porcupine plot.

To assess the individual amino acid contribution to the total free energy profile in a simulation between the wild and L245P mutant, the interaction energy for the previously mentioned residue was carried out using GROMACS package by principle component analysis (PCA). As shown in Figure 8, energy distribution is a centralized form, indicating the overall conformational stability of the system. However, the highest mobility and fluctuation were observed in the L245P mutant, which was depicted as the higher FEL. The lowest energy for the wild type system was found to be 0.113 kcal/mol (Figure 8A), while the mutant variant was 0.605 kcal/mol (Figure 8B). These figures show that the L245P mutation affected the overall conformational stability of the system. These energy levels induced instability in the general structure of the protein. Thermodynamic profile analysis in Figure 8 indicates that longer population of conformations had higher energy in the L245P-mutant (0-14.5 kcal/mol) compared to native type. This observation is consistent with previous PCA scatter and the porcupine plot, further supporting that the L245P mutant decreases the stability.

## DISCUSSION

In this genetic analysis study, we performed TES arrays to identify the genetic defect in a Chinese family with multi-cases of STGD4-like MD retinal degenerations (M107). Using this approach, we successfully identified a novel c.734T > C (p.L245P) PROM1 heterozygous mutation. Our findings highlighted that the variant in the *PROM1* gene was likely a deleterious and disease-causing mutation in this M107 family, thereby expanding the PROM1 mutation spectrum for STGD4-like MD. Then, the protein structure-based virtual screening analysis was performed to identify the protein structure and function of c.734T > C (p.L245P) mutant. Furthermore, atomic level study was performed by molecular dynamic simulations to better understand the effects of the deleterious variant on p.L245P in active site domain of PROM1. The computational prediction methods’ results clearly showed that a novel PROM1 heterozygous mutation significantly affects the protein structure and function, including fluctuation, flexibility, atomic density distribution, and stability.

Despite extensive investigations on STGD4-like MD, the details of its etiology and pathogenesis remain unclear. Familial, identical, and adoption studies have consistently recommended that genetic variation is the most important general factor, which would be an indicator of its high heritability [2, 24-29]. Clinical variability exists in the STGD-like MD, even the same gene of different point mutations can cause variance phenotypes [30]; importantly, different genes caused the STGD-like MD, examples include families with *ELOVL4* mutation, *ABCA4* mutation, and *PROM1* mutation [4, 5, 9, 10, 12, 18-20, 22]. In general, the STGD4-like MD is juvenile-onset MD that is generally related to an *ELOVL4* mutation following *PROM1*. But, most STGD-like MD subjects maintained normal vision until the sixth decade of life. This gradually progressive disease is diagnosed with photophobia, paracentral scotoma, slow dark adaptation, and loss of color and central vision. Symptoms and prevalence of STGD-like MD are the same in altered individuals; members of the same family can show variations in the course of the illness [11]. Clinically, it is hard to predict or screen exactly when the disease will be evident and how fast it will develop, according to marked phenotype variability. It is well established that TES is a cost-effective and an expedited approach to screen and identify pathogenic mutations responsible for inherited disorders on a large scale in comparison to other genetic methods [31, 32]. This is a predominantly accurate technique when the study conditions are particularly heterogeneous, as in a STGD-like MD [33]. By helping to advance imaging techniques, we have been able to further characterize this disease [34].

For the first time ever, this study characterized the novel pathogenic mutation in three-dimensional protein structure, analyzing this change at the structural level. Our findings show that c.734T > C (p.L245P) is functionally damaging and the disease-causing mutation in protein; therefore, we introduce it as a candidate for further clinical precision diagnosis and interpret that the same gene mutation leads to varying onset at different ages, a characteristic that is clinically useful for early prevention.

It has been widely accepted that the computational and theoretical methods such as molecular dynamic simulation, PCA, FEL, and RINs analysis (with parameters of molecular dynamic to describe motion, stability and intercellular binding, respectively) can be used to detect and investigate the effects of lethal polymorphisms on the function and structure of protein [35-40]. Understanding protein conformational changes properly can elucidate the mechanisms underlying MD disease phenotypes for designing suitable drugs and medical managements. In this study, these methods clearly explained the impact of the mutation on the active site of the PROM1 protein, before undertaking further validation by experimental methods. Mutation of the residue alters the overall active site energy by ∼ +6.25 kcal/mol and affects the landscape of the protein and atomic interaction of key residues. The different molecular dynamic parameters such as RMSD, RMSF, DSSP, and Rg index confirmed that the p.L245P pathogenic mutation changes the molecular stability and flexibility as well as the amino acid interaction network and 3D conformational landscape. Interestingly, hydrogen bond interactions and van-der Waals forces of Pro245 with His242, Val73, Asn69, Leu248, Glu249, and Ile241 in the α-helical structure of bHLH-Zip domain significantly increased to maintain a stable contact of the mutant form of PROM1, whereas total protein stability was reduced due to fluctuation in the hinge region of the mutated structure. The substitution of deleterious L245P mutation might change the electrostatic charge distribution in proteins and affect normal interactions. From this analysis, we suggest that the mutated PROM1 domain has drastically decreased functionality and stability capacity as a consequence of the partial unfolding of the α2/α3 helices in the bHLH-Zip domain. This mutation led to a change of a neutral amino acid residue, Leu, to a nonpolar charged residue, Pro, located at the basic repeating unit of the bHLH-Zip. The Leu position in bHLH-Zip plays an essential role in transcriptional regulation of PROM1 [41]. It is likely that the substitution may impair protein stability and proper function, leading to the STGD4-like MD disorder [42]. The Pro mutation in the bHLH-Zip domain may destroy the PROM1 transportation [43], a phenomenon possible and consistent in our patient’s observation. Consequently, this instability and inflexibility affected the main function of PROM1 in the plasma membrane invaginations and disk malfunction, trapping it in the myoid region of the photoreceptors. Functionally, the PROM1 protein is necessary for disk formations in the photoreceptor cells. PROM1 has an influence on the formation and organization of disk within the photoreceptors. Mutations in *PROM1* gene would trap PROM1 in the myoid region of the photoreceptors, preventing it from migrating to the site that formed disks. Accordingly, abnormalities in PROM1 positions in the photoreceptors cells will lead to disk malfunction. Previous studies have shown that different mutants undergo different misfolding pathways and exhibit different pathogenesis according to the different MD-related diseases [10, 12, 18-20]. In addition to an autosomal recessive PROM1 early-childhood-onset retinopathy, an autosomal dominant later-onset macular retinal phenotype has been documented in some individuals heterozygous for PROM1 mutations [18, 44], including in our study.

To the best of our knowledge, this is first report that identified and characterized a novel c.734T > C (p.L245P) mutation of *PROM1* with STGD4-like MD using the TES and multiple molecular dynamic analyses. The findings clearly confirmed that the variant in the *PROM1* gene is most likely the deleterious and disease-causing mutation in our studied family. While variants with incomplete segregation would be regarded as low to moderated penetrance effectors, complete segregation of a one novel variant (in *PROM1* gene) demonstrates the variants are noteworthy candidates in case-control or population studies; however, it warrants more extensive studies of the PROM1, including in various ethnic groups. Altogether, this study provides compelling evidence that the *PROM1* gene mutation should contribute to the progressive causativeness or susceptibility in patients with STGD4-like MD, as well as defines a new approach into the genetic characterization, precision diagnosis and prevention for STGD4-like MD disease.

## MATERIALS AND METHODS

### Ethical consideration, patient information, and clinical assessment

The study was approved by the Ethical committee of Southwest Medical University according to the Helsinki Declaration (1983 Revision) [45]. The cases were coded, and measurements were made in a blinded fashion by molecularist.

A four-generation, 18-related Chinese patients (M107) with familial STGD4-like MD was recruited. The genetic and full pedigree structures of the subjects are shown in Figure 1A. The patient proband claimed to have eye problems since the age of 42. All patients were diagnosed with STGD4-like MD by an experienced ophthalmologist; we also documented the patients’ inheritance patterns, ethnicity and summary information regarding the numbers of affected subject and members who were available for sampling. We obtained medical history and standard ophthalmologic examinations including best corrected visual acuity measurements, slit-lamp biomicroscopy, and color vision tests in all available family members. In addition, FP, FA (Spectralis; Heidelberg Engineering, Heidelberg, Germany), visual field tests (Carl Zeiss, Germany), and retinal structure were examined by OCT (Carl Zeiss, Germany). ERGs were performed using corneal “ERGjet” contact lens electrodes (RetiPort ERG System; Roland Consult, Wiesbaden, Germany) [45]. The ERGs protocol complied with the standards published by the International Society for Clinical Electrophysiology of Vision.

### DNA sampling

Peripheral blood samples in the pedigree M107 family and unrelated ethnically matched healthy control volunteers with no family history of this disorder were collected. gDNA was extracted from peripheral blood lymphocytes according to the standard phenol-chloroform method [45]. The DNA quality was measured by a NanoDrop-2000-spectrophotometer. High quality intact genomic DNA with optical density ratio of 260/280∼ 1.8 and 260/230 > 1.5 were used for further analysis.

### Design of exome capture panel

Illumina paired-end libraries (Illumina, Inc., San Diego, CA) were generated according to the manufacturer’s sample preparation protocol for gDNA [46, 47],. TES on an affected family member was randomly sheared by sonication into fragments of about 300-500 bp and then hybridized to the precapture libraries, which were quantified (PicoGreen fluorescence assay kit; Life Technologies, Carlsbad, CA); their size distributions were determined by a commercial bioanalytical system (Agilent 2100 BioAnalyzer; Agilent Technologies, Santa Clara, CA). For each capture reaction, fifty pre-captured libraries (60 ng/library) were pooled together. Hybridization and wash kits (Agilent Technologies, Santa Clara, CA) were used for the washing and recovery of captured DNA following the standard manufacturer’s protocol. Captured libraries were quantified and sequenced (Illumina HiSeq 2000; Illumina, Inc.) as 100-150 bp paired-end reads, following the manufacturer’s protocols whitch performed at the BCM-FGI core. More than 99% of the targeted coding exons were covered by at least 10 folds non-redundant sequencing reads.

### Variant filtering and homozygosity mapping

After passed quality control (QC) of the illumina reads ( > 70-80% Q30 data, < 0.5% error rate), Burrows-Wheeler Aligner (BWA) V7.10 was used to access sequence reads to the human reference genome from available public online UCSC database (http://genome.ucsc.edu/), version hg19 (build 37.1). Next, recalibration and local realignment were analyzed using the Genome Analysis Toolkit (GATK version 1.0.5974), and the refined sequencing results were subjected to variant calling using a toolkit (Atlas2). Variant annotation was performed by applying ANNOVAR. Sequencing depth > 4, the estimated copy number ≤ 2, SNP quality > 20 (score 20 represents 99% accuracy of a base call) and the distance between two SNPs > 5 were considered the filtration criteria for candidate SNPs [48]. We first searched for known pathogenic mutations in STGD candidate genes and then variants with a MAF (Minor allele frequency) of less than 5% allele frequency in databases of 1000 Genomes Project (1000genomes release_20100804, http://www.1000genomes.org/). In total, the average of 4000 SNPs and INDELs were found after applying these filters. Subsequently, the phenotype of all cases was considered to be similar. At first approach, our focus was on identifying common variants among affected subjects between families, but we also identified any shared variant related to STGD. Consequently, we searched for variants within STGD-related genes in proband (depicted in Figure 1A with pedigree II:2) separately. The selected variants were further analyzed in respect to whether they are homozygous or heterozygous.

### Mutant confirmation and segregation analysis

For mutant confirmation and segregation analysis, we designed locus-specific primers using the online Primer3 program (http://primer3.ut.ee/) for polymerase chain reaction (PCR) amplification of prioritized variants and direct sequencing, upon the gDNAs of family members. Then, the PCR products were validated and confirmed by Sanger sequencing methods on an ABI 3500DX sequencer (Applied Biosystems Inc., Foster City, CA, USA) with the specific primers. Then, all results were analyzed by Sequencing Analysis v.5.2 software (Applied Biosystems). All analyses were performed with two replicates per sample; a non-reverse transcription control and non-template control for each test. The specific primer sequences of *PROM1* gene variant for Sanger sequencing are listed in Supplementary Table 2.

### Mutation analysis *in silico*

The damaging effects of heterozygous mutations (c.734T > C mutation) on PROM1 protein functions were evaluated *in silico* using online web server Mutation Taster (http://www. mutationtaster.org/) [49], Polymorphism Phenotyping version 2 (PolyPhen-2, http://genetics.bwh.harvard.edu/pph2/) [50], SIFT (http://sift.jcvi.org/) [51], Have Your Protein Explained (HOPE) [52], and PANTHER (http://www.pantherdb.org/tools/csnpScoreForm.jsp) programs [53]. Functional impact of the mutation was documented as ‘‘tolerated’’ or ‘‘damaging’’ for SIFT and as ‘‘polymorphism’’ or ‘‘disease causing’’ for Mutation Taster. PolyPhen-2 classifies predicted effects of amino acid substitutions, marking the function of human proteins as ‘‘benign’’, ‘‘possibly damaging’’, ‘‘probably damaging’’, or ‘‘unknown’’. To predict stability changes upon mutation from the protein sequence or structure, I-Mutant3.0 was used based on the stability predictors (DDG < 0: decrease stability, DDG > 0: increase stability) [54]. Pairwise alignment between template and target were performed by EBI/EMBL. Also, NCBI online data bases (http://www.ncbi.nlm.nih.gov/) were used to analyze the PROM1 conservation in deferent organism gene by inputting the data into the HomoloGene software of NCBI “Show Multiple Alignment” and Jalview software [55].

### Homological based modeling

The sequence of human PROM1 was downloaded from the universal protein resource (http://www.uniprot.org) (Entry: O43490). The template for sequence alignment was identified by searching human PROM1 on PDB using the BLASTp program. The 3D structure of PROM1 (XKD) was created using MODELLER version 9.17 [56], then the model was viewed using PyMol software [57]. The initial coordinates of PROM1, which were used in our molecular simulations, were based on crystal structure of the template protein (PDB Entry: 4wid). A mutation of Leu to Pro at position 245^th^ was generated *in silico* from the wild-type crystal structure using Pymol software [58]. The general streochemical quality of the final modeled protein structure was evaluated using PROCHECK [59], ProSA-web (protein structure Analysis) [60], RAMPAGE, and QMEAN analysis [61].

### Molecular dynamic simulation

Molecular dynamics (MD) simulations investigate the motions and fluctuations of a system containing discrete particles under the impact of internal and external forces. The role of this application base on this method is broad, ranging from atoms to a molecule. The initial 3D structure made from modeler was optimized using molecular dynamic simulation. The 130 ns simulations of p.L245P on the active functional extracellular domain of PROM1 (position 179-433, Supplementary Figure 3) were performed using the GROMCS molecular dynamics package version 5.1.3, which, on the Centos linux system, implements GROMOS96 package version 43a1 force field. pdb2gmx tools from GROMACS software package was applied to generate the topology file. The 3D human PROM1 was surrounded by a cubic periodic box of the simple point charge model of water molecules with a margin of 1.0 Å along each dimension. [62]. In detail, the number of water molecules in the cubic cage was approximately 120,000. Also, the molecular dynamical systematic simulations charges were neutralized by using sufficient Na^+^ and Cl^-^ ions. All covalent bonds to hydrogen atoms were constrained using the SHAKE algorithm. Electrostatic interactions were calculated using the particle-Mesh Ewald algorithm [63] with a cutoff of 10 Å for Lenard-Jones interactions. Periodic boundary conditions were applied to avoid edge effects. Prior to molecular dynamic production, 50,000 steps of steepest-descent minimization were used to the solvent. After energy minimization, the system was equilibrated with NVT (isochoric-isothermal) and NPT (isothermal-isobaric) ensembles under the condition of position restraint for heavy atoms, with 100 ps with gradual heating from 300 K [64]. In addition, stable salt bridges were extracted based on a distance cut-off value of 0.4 nm. The atomic coordinates of each model were saved every 1-ps for the analysis. The temperature and pressure coupling was applied using the modified Berendsen thermostat algorithm [65] at 300 K and Parrinello-Rahman algorithm [66] at 1 atmosphere with a link constant of 0.1 ps and time duration of 100 ps. The comparative analyses were saved every 1 ps time interval for further analyses, including RMSF for all human PROM1 structures. RMSD, secondary structure modifications, Rg, hydrogen bond occupancy, secondary structure analysis, PCA, and clustering analysis were performed for the wild-type and mutated form [67]. Additionally, the structural changes were visualized using VMD version 1.9.2 and Pymol software. The plots were conducted using xmgrace tool and secondary structure analysis, by DSSP of the GROMACS packages, respectively. Furthermore, a 130 ns molecular modeling trajectory of the system was obtained under constant pressure at 300 K using the GROMACS software package.

### Residue interaction networks of the wild- and mutant-type

To analyze the mutatant residue on protein sequence, we used the open source software platform, Cytoscape v3.3.0 [35]. Cytoscape allows us to map the various interactions of wild- and mutant-type residue sources data in RIN models. In this regard, the average structure derived from the 130 ns trajectory of each system, wild type and L245P mutant was used to construct the RINs in 2D graphs using RING 2.0 web server [68]. This is a new version of the RING software for the identification of covalent and non-covalent bonds in protein structure. After determining the interaction between amino acids, RING used several tools to define non-covalent interaction between the amino acids, including hydrogen bonds, salt bridges, and interaction in closest atom. We mapped the information about the protein mutation and natural variation of a PROM1 protein onto all the corresponding nodes in the pathway. All the processed residues (functional extracellular domain of PROM1 in position 179-433) were separately imported into Cytoscape 3.3.0 to reconstruct amino acidic networks using RINalyzer plug-in software [36]. Therefore, the Cytoscape archive files were constructed by submitting the crystal structure of both the wild type and the mutant protein to RING server [37].

### Analysis of molecular dynamic trajectory

The trajectory files were analyzed using gmx rms, gmx rmsf, and gmx gyrate GROMACS utilities to obtain the RMSD, RMSF, and Rg. The numbers of distinct intermolecular hydrogen bonds formed between all residues during the simulation were calculated using gmx hbond utility. Secondary structure analyses using DSSP module were performed for both the wild type and L245P mutant over the whole simulation period.

### Circular dichroism spectroscopy analysis

The secondary structure of the wild-type and p.L245P mutant system were determined by CD spectroscopy during 130 ns modeling using DichroCalc server [69]. CD spectroscopy is a well-established technique for measuring the secondary structure, dynamics, and folding pathways of proteins in the “far-UV” spectral region (190-250 nm); where alpha-helix, beta-sheet, and random coil structures each give rise to a characteristic shape and magnitude of CD spectrum [70].

### Principal component analysis (PCA)

PCA is a method that reduces the complexity of the data and extracts the concerted motion that are essentially correlated and presumably meaningful for biological functions during simulations. PCA was performed to determine the correlated motions of the residues to a set of linearly uncorrelated variables named principal components that are significant for the biological function of the protein during the course of simulation [38]. In PCA analysis, a variance/covariance matrix was constructed from the trajectories after the removal of the rotational and translational movements. PCA is a widely used protocol to simplify eigenvectors and eigenvalues of bio-molecules from the molecular modeling trajectories by relating it to the dimensional reduction method. GROMACS utility tools were used to perform PCA, with using normal mode wizard of VMD. PCA scatter plots were then created by the xmgrace program.

### Free energy landscape (FEL)

FEL promotes the dynamic energy distribution and structure-function correlation of mutational residues variable in the protein system, which helps to visualize the stability of wild and mutant conformations for a protein [39]. The free energy minima regularly characterizes the conformational group in the stable states. The free energy barriers represented by the transient states of free energy values of backbone atoms in different systems, according the Gibb’s free energy method [40]. In this study, we compared FEL values to identify the dominant conformational states, function of the enthalpy, and entropy of protein in wild and mutant conformations of PROM1 protein by using GROMACS package software based on the PCA data.

## SUPPLEMENTARY MATERIALS FIGURES AND TABLES



## Abbreviations

STGD: Stargardt disease
MD: macular degeneration/dystrophy
adSTGD: autosomal dominant trait of STGD
PC: principal component
FEL: Free energy landscapes
FA: fundus autofluorescence
ERGs: Electroretinograms
OCT: optical coherence tomography
TES: targeted exome sequencing
SNP: single nucleotide polymorphism
INDELs: Insertions/Deletions
MAF: minor allele frequency
p.L245P: Leu at residue 245
bHLH-Zip: basic helix-loop-helix leucine zipper domain
SIFT: scale-invariant feature transform
QMEAN: quantitative model energy analysis
RMSD: root mean square deviation
ns: nanosecond
RMSF: root mean square fluctuation
DSSP: defined secondary structure of protein
CD: Circular dichroism
gDNA: genomic DNA
RINs: residue interaction networks
PCA: principal component analysis
BWA: Burrows-Wheeler Aligner
GATK: Genome Analysis Toolkit
PCR: polymerase chain reaction
HOPE: Have Your Protein Explained
