# Supplementary material for: Genetic identification and molecular modeling characterization reveal a novel PROM1 mutation in Stargardt4-like macular dystrophy
Source: Oncotarget. 2017 Nov 9;9(1):122–41. doi: 10.18632/oncotarget.22343 (PMC5787432; doi:10.18632/oncotarget.22343)
Supplement: Supplementary file 1 [file oncotarget-09-122-s001.pdf]

Genetic identification and molecular modeling characterization reveal a novel *PROM1* mutation in Stargardt4-like macular dystrophy

SUPPLEMENTARY MATERIALS

Active Residues in Wild:

3, 18, 19, 20, 21, 56, 58, 59, 60, 63, 66, 67, 68, 70, 71,
72, 74, 75, 76, 77, 78, 79, 80, 81, 83, 85, 135, 136, 137, 138,
139, 163, 164, 165, 166, 167, 169, 171, 173, 174, 175, 177, 180, 181,
182, 202, 235, 236, 238, 242, 243, **245**, 246, 248, 249, 250, 254.

Active Residues in L245P Mutant:

2, 3, 5, 6, 14, 18, 20, 21, 22, 25, 43, 45, 67, 101, 129,
135, 136, 137, 138, 139, 140, 158, 159, 160, 161, 162, 164, 165, 167,
168, 169, 170, 171, 172, 173, 174, 175, 177, 178, 179, 180, 182, 183,
187, 188, 194, 195, 197, 198, 200, 201, 202, 204, 207, 248, 252, 253.

Supplementary Figure 1: The active functional extracellular domain residues of PROM1 position in the wild type and L245P mutant systems.

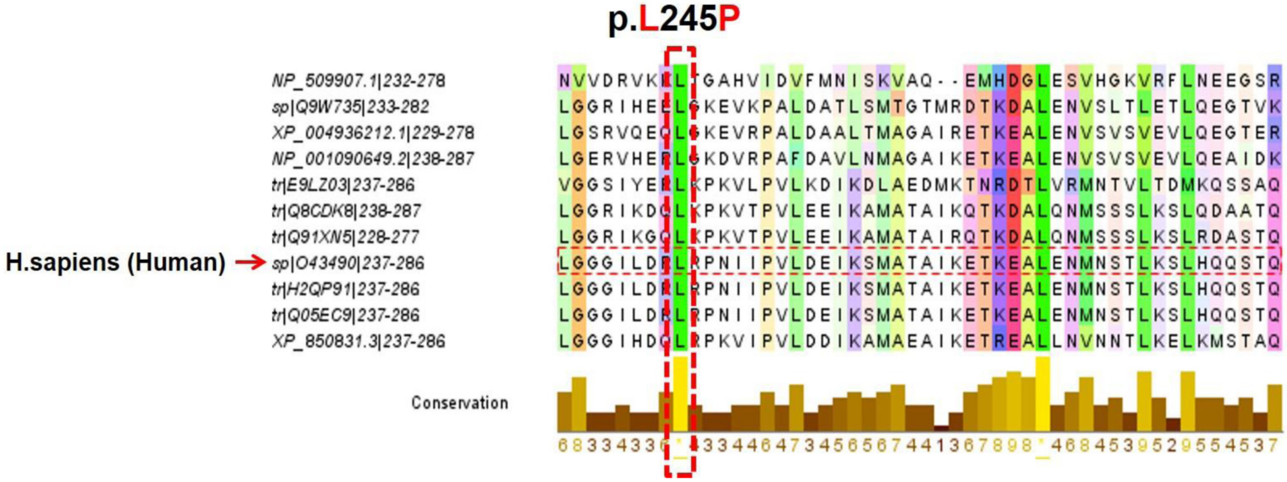

Supplementary Figure 2: Conservation analysis of the PROM1 p.Leu 245 amino acid residue. The high light amino acid residue Leu. (Leucine) in green is conserved in different species. Computer-based protein analysis indicates that the variant in the PROM1 was likely deleterious and the disease-causing mutation in our family. The protein accessions and their species information of organisms and were listed in the right side.

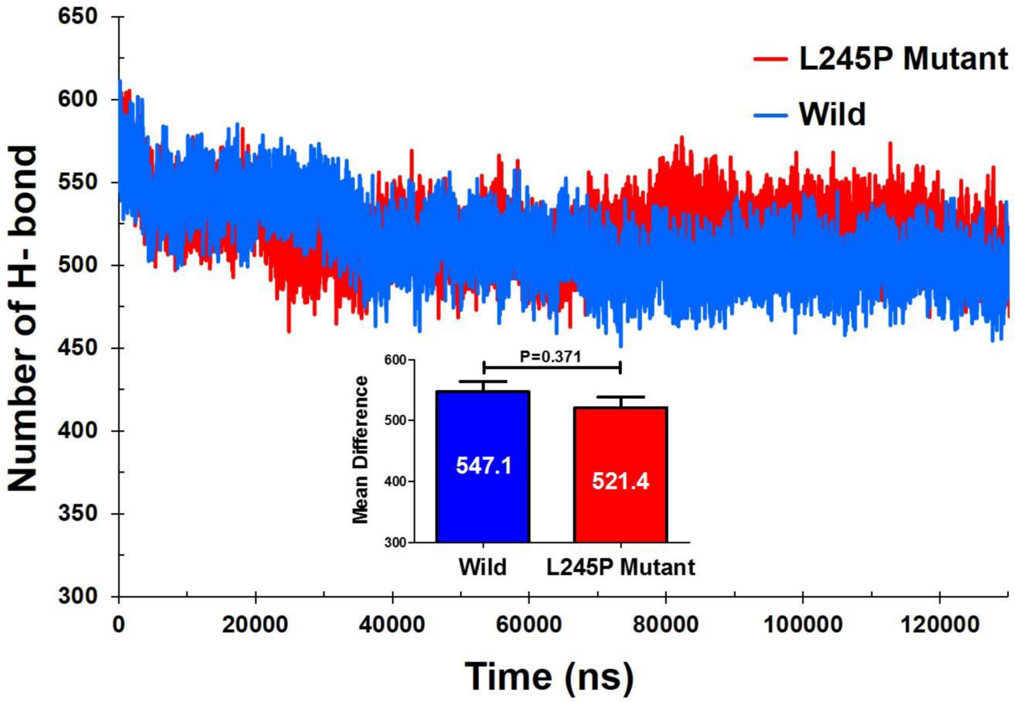

Supplementary Figure 3: The average number of total hydrogen bonds of PROM1 protein during 130-ns of molecular dynamic simulation. These results show that L245P point mutant proteins had different fluctuation patterns and different number of H-band from the wild-type protein, with significant high fluctuations and low stabilizing.

Supplementary Table 1: Specific sequences of PROM1 primers, PCR product size.

Target gene	Accession No.*	Sequence (5'→3')	Length	Tm (°C)	Size (bp)	GC%
PROM1 (M107)	NM_006017	F, TCCAACGACTCAACTCTGCC	19	60.00	506	50.00
		R, GGAGAGAATGAGGGAGGGGT	19			60.00

Abbreviations: PROM1, prominin (mouse)-like 1 protein; F, forward primer; R, reverse primer; Tm, optimum primer melting temperatures; GC%, guanine and cytosine percentage.
*Genbank accession number of cDNA and corresponding gene, available at http://www.ncbi.nlm.nih.gov/

Supplementary Table 2: PROCHECK, ProSA-web, and QMEAN analysis of the final models of the wild and mutant structures.

Quality checks	Generated model's name		
	4wid	Before mutation	p.L245P
**ProSA-web**			
Z-score	-9.67	-4.31	-4.15
**QMEAN**			
QMEAN Z-score	-2.09	0.442	0.369

All amino acid abbreviated according the International Union of Pure and Applied Chemistry (IUPAC). The possible effects of the mutation on proteins structural predicted upon the RefSeq and UCSC genome databases. 4wid: template; p. L245P, mutant protein at residue 245.
